# A method to alleviate false-positive results of the Elecsys HIV combi PT assay

**DOI:** 10.1038/s41598-020-80047-0

**Published:** 2021-01-13

**Authors:** Xiaolan Lu, Minghong Zhang, Wen Liu, Nan Sheng, Qin Du, Maoxin Zhang, Xiaolan Guo, Guangrong Wang, Qiang Wang

**Affiliations:** 1 Department of Laboratory Medicine, Affiliated Hospital of North Sichuan Medical College, Nanchong, Sichuan People’s Republic of China; 2 College of Laboratory Medicine, North Sichuan Medical College, Nanchong, Sichuan People’s Republic of China; 3 Faculty of Laboratory Medicine, Center for Translational Medicine, North Sichuan Medical College, Nanchong, Sichuan People’s Republic of China; 4 Nanchong Center for Disease Control and Prevention, Nanchong, Sichuan People’s Republic of China

**Keywords:** Immunological techniques, Assay systems, HIV infections

## Abstract

To explore the effects of urea dissociation on reducing false-positive results of  the Elecsys HIV combi PT assay. A retrospective analysis was used to evaluate the false-positive rate of the Elecsys HIV combi PT assay. Six false-positive sera, six positive sera and six sera from patients with early HIV infection were collected. Dissociation was performed using 1 mol/L, 2 mol/L, 4 mol/L, 6 mol/L, or 8 mol/L urea, and HIV screening assay were then detected to select the appropriate concentration of urea dissociation. Next, 55 false-positive sera and 15 sera from early HIV infection were used to verify the best concentration of urea to achieve dissociation. Retrospective analysis showed that the COI of the Elecsys HIV combi PT assay in false-positive sera ranged from 1.0 to 200.0, and approximately 97.01%(227/234) of false-positive sera were in the range of 1.0–15.0. The avidity index (AI) in positive and false-positive sera decreased as the urea dissociation concentration increased. When the dissociation concentration was 6 mol/L, the AI of false-positive serum was between 0.0234 and 0.2567, and the AI of early HIV infection sera was between 0.4325 and 0.5017. The difference in AI between false-positive and positive samples was significant. When negativity was defined as an AI of less than 0.3970, the sensitivity and specificity were 100.0% and 100.0%, respectively. Urea-mediated dissociation could significantly reduce the false-positive rate of the Elecsys HIV combi PT assay with a low COI. Our findings provided a reference for distinguishing positive and false-positive of the Elecsys HIV combi PT assay.

## Introduction

Human immunodeficiency virus (HIV) infection is a serious problem affecting human health^1,2^. Since the first case of HIV infection was discovered in 1981, the epidemic of HIV infection has spread worldwide^3^, and no treatments have currently been developed that can completely eradicate HIV^1,4,5^. Therefore, early identification, treatment, and self-management are often the most effective means to prevent the spread of HIV. At present, the most important method for the diagnosis of HIV infection is the detection of anti-HIV antibodies, which is typically accomplished using screening tests (primary screening and retest) and confirmatory tests^6^. Rapid and accurate detection of anti-HIV antibodies could provide a reliable basis for clinical diagnosis of HIV infection.

Anti-HIV antibody screening uses highly sensitive reagents to detect all potential HIV infected candidates. To date, the screening methods for detection of anti-HIV antibodies include enzyme-linked immunosorbent assays (ELISAs), chemiluminescence assays, electrochemiluminescence assays, immunofluorescence, rapid detection, and other tests^6–11^. Electrochemiluminescence assays show high sensitivity, are simple to operate, and yield quick results^7,10^, making them suitable for simultaneous detection of large quantities of specimens.

However, interfering factors, such as pathological diseases and the high sensitivity of electrochemiluminescence assays, increase the false-positive rate of anti-HIV antibody detection by electrochemiluminescence assays^7,10,12–15^. Notably, false-positive results cause huge wastes in medical resources, and have many negative effects on society^16,17^. In addition, false-positive anti-HIV antibodies are mainly excluded using confirmatory tests and nucleic acid detection, which are time-consuming and expensive^6,15,18^. Thus, it is necessary to develop a simple, convenient, and economical assay to identify false-positive results in anti-HIV antibody detection.

Urea is a type of dissociating agent that, functions to dissolve and denature protein. Previous studies in our laboratory have found that urea-mediated dissociation alleviates the false-positive *Treponema pallidum*-specific antibodies detected by ELISA^19^. However, whether urea-mediated dissociation can reduce the false-positive rate of the Elecsys HIV combi PT assay has not been studied.

Therefore, in this study, we evaluated and validated the dissociation of urea in an attempt to alleviate reduce false-positive of the Elecsys HIV combi PT assay.

## Results

### Retrospective study results

**Figure 1:**
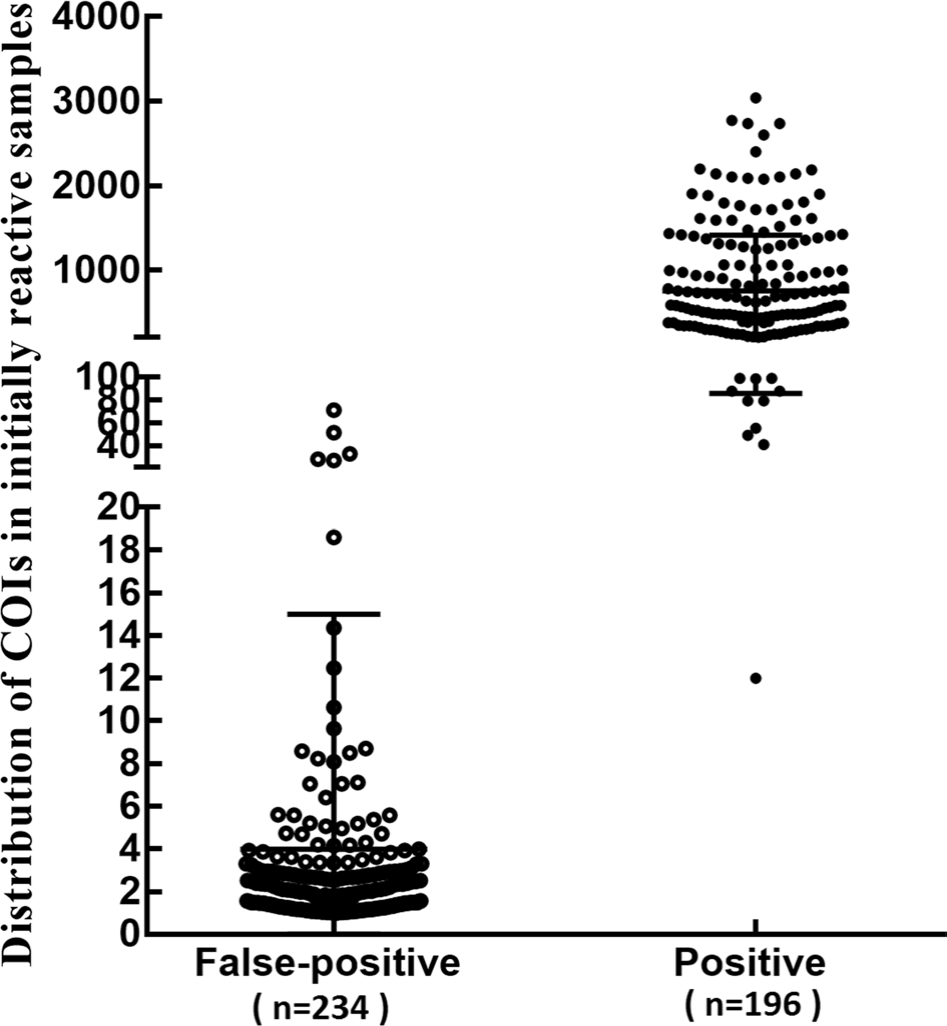
Distribution of screening values of COIs in false-positive and positive specimens.

From January 1, 2018 to June 30, 2019, 129,628 outpatients and inpatients (ages 0–92 years) attending the affiliated Hospital of North Sichuan Medical College were screened for HIV infection. In total, 640 positive cases were detected by primary screening, 210 cases of which were not assessed by confirmation tests. Through re-examination tests, confirmation tests, and follow-up confirmation tests, 196 cases were confirmed to be HIV-1 positive, only one of which was early HIV infection. Additionally, 234 cases were negative (false-positive). The value of COI in false-positive serum was typically between 1.0 and 15.0, whereas that in positive samples was typically over 100.0 (Fig. 1).

When the COIs of the Elecsys HIV combi PT assay were in the ranges of 1.0–15.0, 15.0–50.0, 50.0–100.0, 100.0–200.0, and greater than 200.0, the false-positive rates were 99.56%, 66.67%, 20.0%, 3.23%, and 0.0%, respectively, and the corresponding positive rates were 0.44%, 33.33%, 80.0%, 96.77%, and 100.0%, respectively. The overlap range of COI of positive and false-positive specimens was mainly from 1.0 to 200.0 (Table 1).

**Table 1:** Distribution of screening values of COIs in false-positive and positive specimens.

COI	N	Confirmed by western blotting	
		Positive [n (%)]	Negative [n (%)]
1.0–15.0	228	1 (0.44%)	227 (99.56%)
15.0–50.0	6	2 (33.33%)	4 (66.67%)
50.0–100.0	10	8 (80.00%)	2 (20.00%)
100.0–200.0	31	30 (96.77%)	1 (3.23%)
> 200.0	155	155 (100.0%)	0 (0.0%)
Total	430	196	234

*COI* cut-off index.

### Laboratory features of study subjects

**Figure 2:**
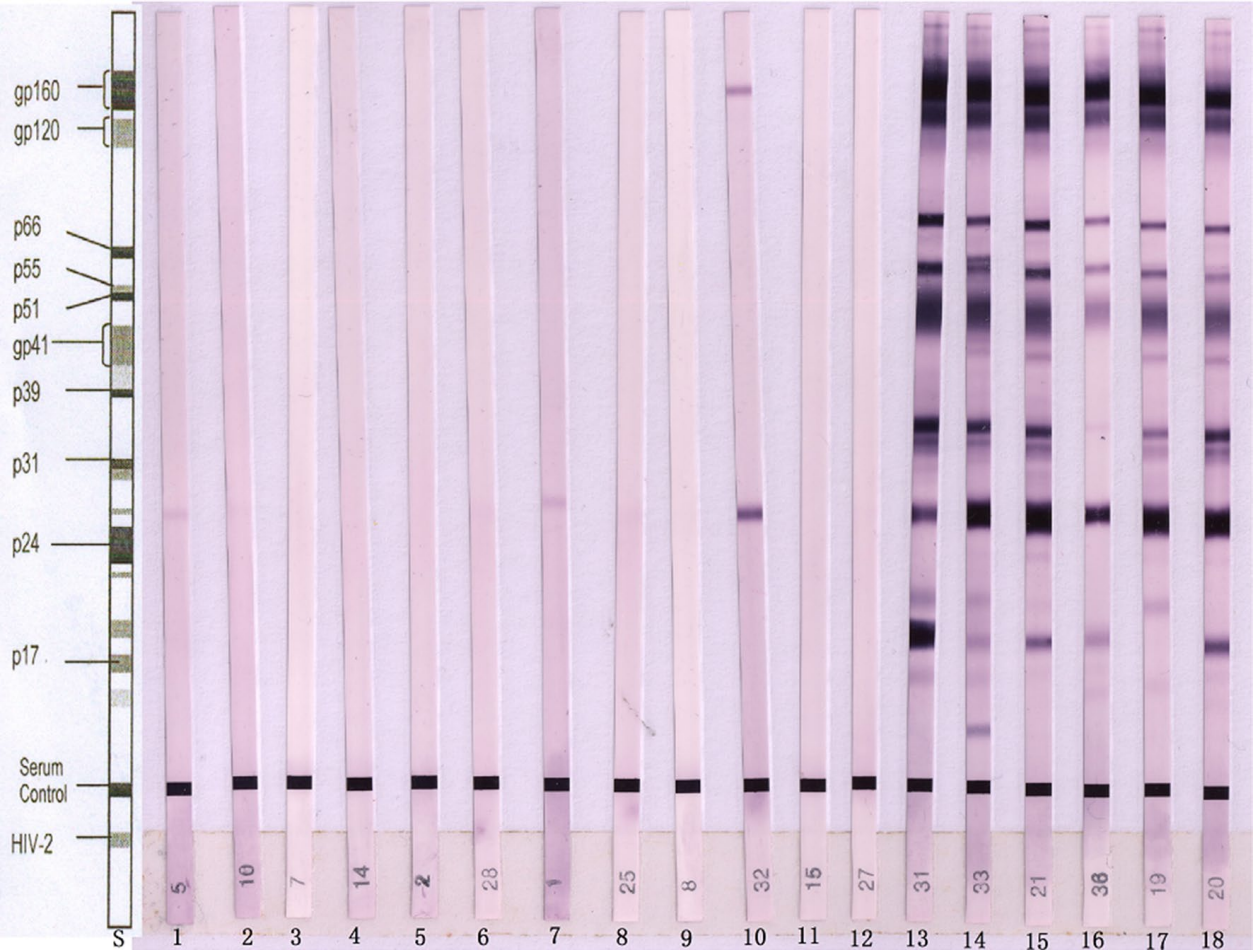
First western blot analysis of human sera for urea dissociation tests. *S* standard line. Lanes 1–6: sera obtained from patients with false-positive results, showing no bands or only the P24 band for specific antigens of HIV-1. Lanes 7–12: sera obtained from patients with early HIV infection, showing no bands or only one distinct band for specific antigens of HIV-1. Lanes 13–18: sera obtained from patients with positive results, showing more than two distinct bands for specific antigens of HIV-1.

**Figure 3:**
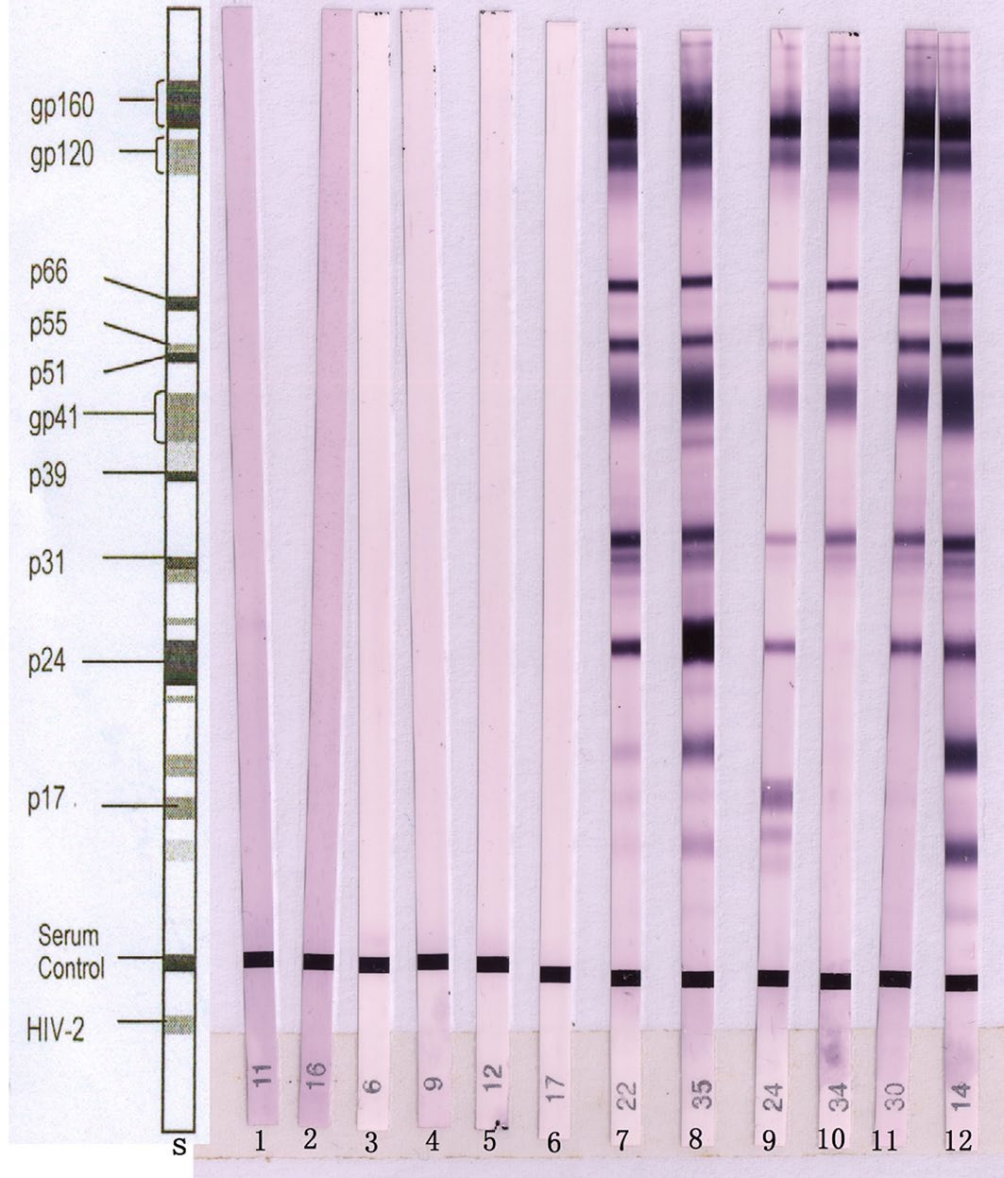
Western blot analysis of false-positive results and early HIV infections after follow-up for 2 weeks to 3 months. *S* standard line. Lanes 1–6: sera obtained from patients with false-positive results, showing no bands or only a P24 band for specific antigens of HIV-1. Lanes 7–12: sera obtained from patients with early HIV infection, showing more than two distinct bands for specific antigens of HIV-1.

Serum samples that were positive in the initial screening were collected. Six serum samples were false-positive; these samples were subsequently confirmed to be negative or only positive for P24 (Fig. 2, lanes 1–6) by initial confirmatory tests and were still negative or only positive for P24 (Fig. 3, lanes 1–6) by follow-up confirmatory tests 3 month later. Additionally, six serum samples were positive and confirmed to be positive (Fig. 2, lanes 13–18) by confirmatory tests. Six cases of early HIV infection were negative or positive for P24 and gp160 (Fig. 2, lanes 7–12) by initial confirmatory tests and were confirmed to be positive by follow-up tests 2 week to 3 month later (Fig. 3, lanes 7–12).

In addition, 70 serum samples that were positive by primary screening were collected for verification experiments. Among them, 55 false-positive serum samples were confirmed to be negative by initial confirmatory tests and follow-up confirmatory tests. Moreover, 15 serum samples were confirmed to be negative or indeterminate by initial confirmatory tests and were confirmed to be positive by follow-up confirmatory tests 2 week to 3 month later. All results are shown in Table 2.

**Table 2:** Results of 88 serum samples detected by different methods.

Study participants	N	COI	Initial Western blotting
False-positive sera	6	5.27 ± 3.16	Negative or indeterminat
Positive sera	6	251.27 ± 139.20	Positive
Early stage infection sera	6	6.63 ± 3.96	Negative or indeterminat
Validation experiment			
False-positive sera	55	6.31 ± 3.21	Negative or indeterminat
Early stage infection sera	15	7.42 ± 4.35	Negative or indeterminat

*COI* cut-off index.

### Selection of the best dissociation concentration of urea

The AIs of all samples decreased as the urea dissociation concentration increased. When the concentration of urea was 1 mol/L, there was significant difference in AI between false-positive group and positive group (*P* < 0.05), but there was no significant difference in AI between false-positive group and early HIV infection group (*P* > 0.05). When the concentration of urea was 2 mol/L and 4 mol/L, the AI of false-positive samples was significantly lower than that of positive group and early HIV infection group (*P* < 0.01), but there was no significant difference in AI between positive group and early HIV infection group (*P* > 0.05). When the concentration of urea was 6 mol/L and 8 mol/L, the AI of false-positive group was significantly lower than that of positive group and early HIV infection group (*P* < 0.001), and the AI of early HIV infection group was lower than that of positive group (*P* < 0.05).

**Figure 4:**
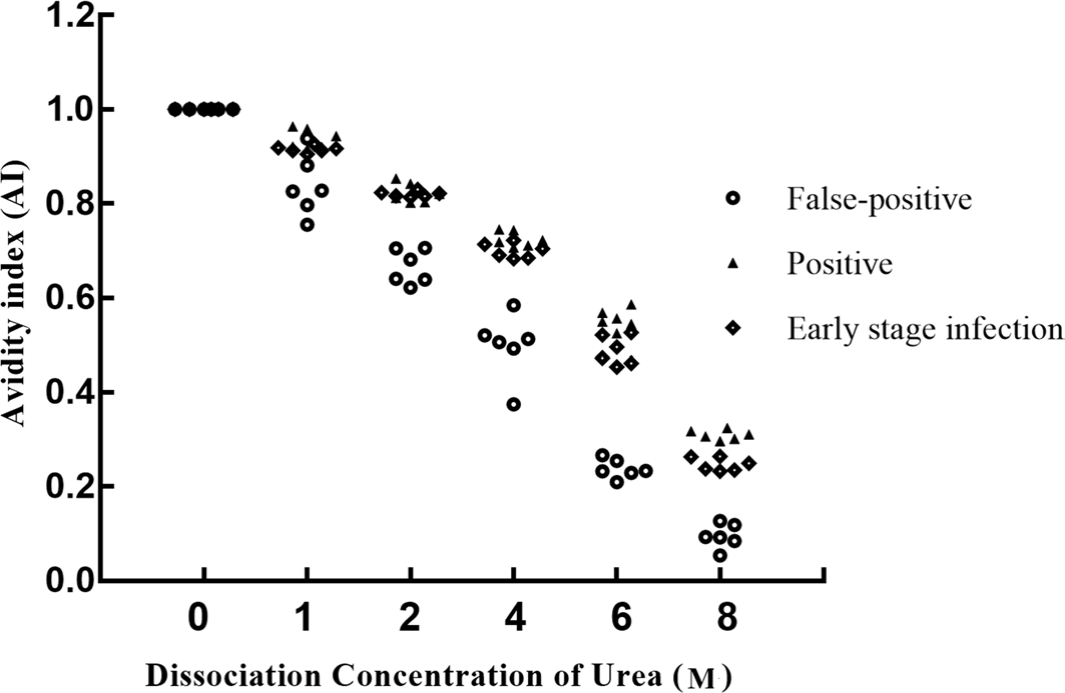
Urea dissociation test to determine the appropriate dissociation concentration.

The degree of decline of AI in the false-positive group was significantly faster than those in the positive group and the early HIV infection group. When the urea dissociation concentration was 6 mol/L, a differential effect was observed between positive and false-positive results (Fig. 4).

### Verification of the best dissociation concentration of urea

**Figure 5:**
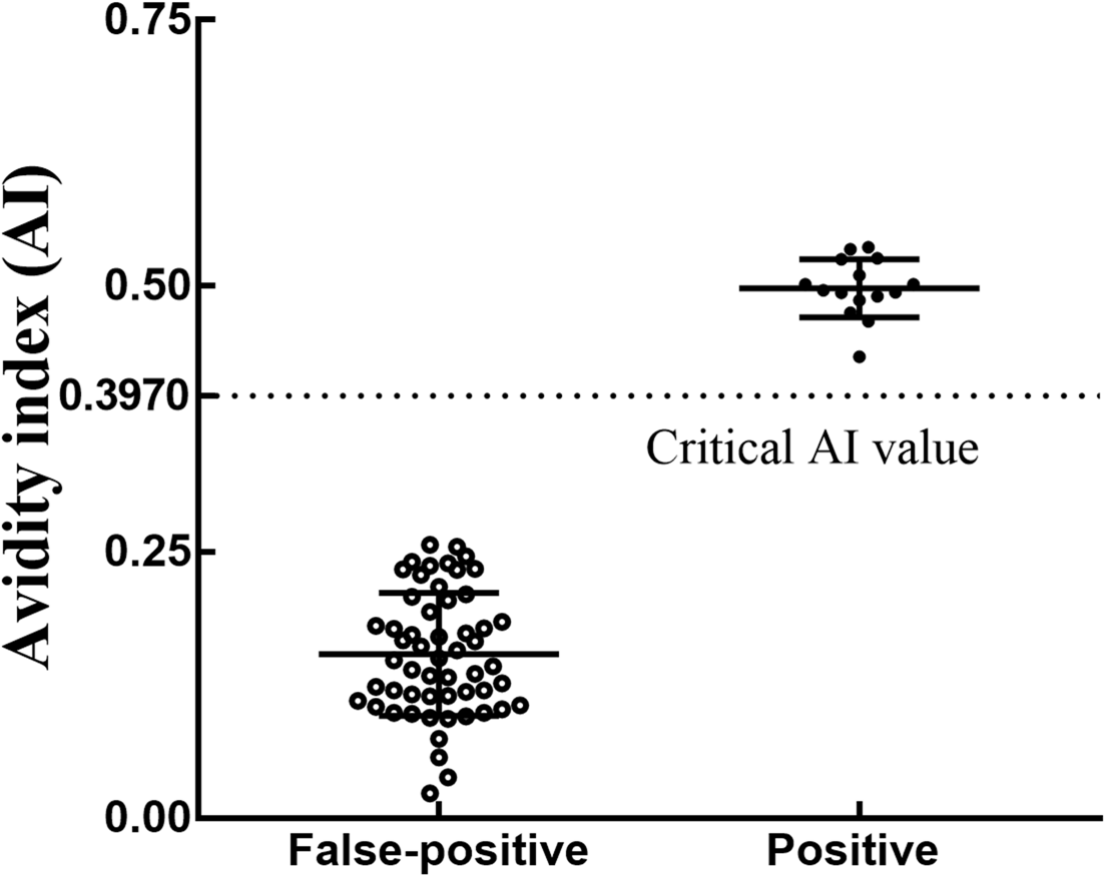
Validation experiment for 6 mol/L urea. After dissociation with 6 mol/L urea, the AI of false-positive serum samples was measured, and the sensitivity and specificity values were determined.

According to the function NORMDIST, the AI at 0.01% was 0.3970, which was the set critical value. When negativity was defined as an AI of less than 0.3970, after dissociation with 6 M urea, the AIs of 55 false-positive sera were between 0.0234 and 0.2567, indicating that the specificity was 100.0% (95% confidence interval 91.87%, 100.0%); and 15 sera of early HIV infection were between 0.4325 and 0.5017, indicating that the sensitivity was 100.0% (95% confidence interval 87.36%, 100.0%) (Fig. 5).

## Discussion

Fourth-generation HIV screening assays detected by electrochemiluminescence analysis have been used in many laboratories^20^, which greatly improved the sensitivity of detection, shortened the time required for detection, and had positive effects on the diagnosis, treatment, and management of HIV infection. However, interference factors (such as pathological factors and biological factors) and the increased sensitivity of the detection method resulted in higher false-positive of Elecsys HIV combi PT assay^7,10,12–15^. Therefore, it is necessary to develop an improved electrochemiluminescence assay to eliminate interference and reduce false-positives.

In this study, 196 HIV-1-positive cases were confirmed by primary screening tests, confirmatory tests, and follow-up confirmatory tests from January 1, 2018 to June 30, 2019. Interval distribution analysis of the results of primary screening tests for HIV-1 showed that as the COI value of the primary screening test increased, the probability of diagnosis of HIV infection was higher. These results were consistent with the results of Jensen et al.^21^, who showed that the positive predictive value of HIV-1 infection increased as the S/CO ratio increased. Importantly, increasing the threshold of screening tests would result in missed diagnoses.

A retrospective analysis of 234 false-positive cases, which were confirmed as negative, showed that the median COI value in false-positive serum was much lower than that in positive serum. The value of the COI in primary screening tests ranged from 1.0 to 200.0, and approximately 97.01% of false-positive samples were in the range of 1.0–15.0. These findings were consistent with a report by Liu et al.^20^. Moreover, when Elecsys HIV combi PT assay was detected by electrochemiluminescence assays, the COI value in false-positive serum samples ranged from 0.9 to 14.9 in approximately 94.59% of cases. Thus, the false-positive rate of the Elecsys HIV combi PT assay was high, and the COI value of false-positive sera was between 1.0 and 15.0.

According to our HIV screening strategy, once the initial screening was positive, the patient must undergo a retest and confirmatory test to determine the final HIV infection status^6^. Most cases with a high COI value (e.g., COI > 200.0) could be diagnosed directly through the first western blotting confirmation test. However, for cases with low COI values (e.g., COI = 1.0–15.0), most were false-positive, and a few had early HIV infections. It is difficult to distinguish false-positives and early HIV infections using western blotting because false-positives may also show positivity for the P24 antigen or other single antibody band caused by crossreaction; the patient must be followed up for 1, 3, or even 6 months to reconfirm the results^6,8^, and this can waste a lot of manpower, materials, and financial resources. Moreover, there may be a delay in the diagnosis and treatment of the patient, which could cause a heavy psychological burden to the patient^16,17^. Parker et al.^15^ reported that polymerase chain reaction (PCR) testing could be directly used in serum with an S/CO of less than 4.0. However, PCR-based nucleic acid testing is expensive and requires certain technology and equipment^6,18^, which may limit its applications in developing countries, such as China. Therefore, in order to avoid time-consuming and expensive confirmatory tests, it is necessary to establish a timely and cost-efficient method to eliminate interference of false-positives caused by crossreaction, ensure the high specificity of detection, and maintain high detection sensitivity.

Because urea functions to dissociate proteins, the AI decreased as the urea dissociation concentration increased. This effect was likely related to the increase in the denaturant (urea) concentration and denaturation degree. When selecting control serum samples, we selected positive cases with high and early HIV infection with a low COI. In the early stages of HIV infection, the virus replicates in large quantities, and few antibodies are produced^22–26^. P24 antigen is the core HIV-1 protein and is present in the blood before the production of anti-HIV antibodies and changes synchronously with nucleic acid levels during the early stages of HIV infection^27–31^. Thus, the AI of early HIV infection was significantly higher than that in false-positive samples after urea dissociation.

The principle of selecting the urea dissociation concentration was to choose the urea concentration with the most obvious difference between the false-positive and true-positive samples according to the AI of sera. When the dissociation concentration was 1 mol/L, there was no significant difference in AI between true-positive and false-positive sera. When the dissociation concentrations were 2 mol/L, 4 mol/L, and 6 mol/L, the AIs of false-positive samples were significantly lower than those in positive and early HIV infection samples; however, when the dissociation concentration was 6 mol/L, the difference in AI between early HIV infection and false-positive samples was the most significant. Additionally, when the dissociation concentration was 8 mol/L, the denaturation ability was the strongest, and the protein was denatured completely. Therefore, a dissociation concentration of 6 mol/L was considered optimal and could properly distinguish positive and false-positive results.

Because the serum AI of the early HIV infection group was lower than that of the positive group when the urea concentration was 6 mol/L, the early HIV infection serum of was selected for the verification experiment. When the urea dissociation concentration was 6 mol/L, the AIs were significantly different between early HIV infection and false-positive samples, indicating that early HIV infection and false-positive samples could be distinguished when using a urea concentration of 6 mol/L. When the critical value of AI was 0.3970, approximately 0.002% of false-positive samples were regarded as positive; in contrast, when the COI value was between 1.0 and 15.0, 99.56%(227/228) of the false-positive samples were regarded as positive. Accordingly, the false-positive rate was greatly reduced. Moreover, for the positive sera, only approximately 0.01% of positive samples were misjudged as negative.

Owing to the difficulty of collecting early HIV infection samples, only 15 cases were collected for the verification experiment; so the results of this study still have some limitations. However, it can be learned from the dissociation experiment that the experimental conditions resulting from the screening results basically would not cause a false-positive result. Thus, to a certain extent, urea dissociation tests could help to distinguish the authenticity of positive serum with low values. However, additional studies are required to confirm these findings. Therefore, individuals at high risk should be followed up after epidemiological investigation because the probability of a false negative is approximately 0.01% (1 in 10,000).

In summary, in this study, we developed an electrochemiluminescence-based assay for the Elecsys HIV combi PT assay using the urea dissociation method. Our results showed that the false-positive rate of Elecsys HIV combi PT assay was high but that the approach could be used to screen samples with a low COI (e.g., when the COI ranged from 1.0 to 15.0) owing to the use of urea dissociation.

## Materials and methods

### Screening system and retrospective analysis

Elecsys HIV combi PT assay was detected by electrochemiluminescence assay (Cobas E602, Roche, Inc., Germany). Results for the Elecsys HIV combi PT were given as in the form of a cut-off index (COI) and considered to be positive if the COI ≥ 1.0.

The laboratory information system (LIS) of the Affiliated Hospital of North Sichuan Medical College (Sichuan China) was used to retrieve the data of patients with HIV antibody screening test from January 1, 2018 to June 30, 2019. The cases whose test results were greater than or equal to 1.0 were collected, and the patient information and the results of HIV confirmation test were analyzed retrospectively. Cases without confirmatory experiment were excluded.

The positive results of screening can be further divided into false positive, indeterminate and true positive by re-examination and confirmation test. For indeterminate or negative WB results by the first confirmation test, then another WB tests were performed after 2 weeks to 3 months to determine whether they were infected with HIV.

### Study participants

Eighty-eight serum samples were collected from outpatients and inpatients of the affiliated Hospital of North Sichuan Medical College for HIV screening tests and Nanchong Center for Disease Control and Prevention for western blotting. Six false-positive sera, six positive sera and six sera from patients with early HIV infection were used in urea dissociation tests to select the appropriate urea dissociation concentration. The COIs of six false-positive sera and six early HIV infection sera was between 1.0 and 15.0; and six positive sera was more than 200.0. All cases were confirmed by initial western blotting and follow-up western blotting.

Additionally, 70 samples (55 false-positive sera and 15 early HIV infection sera) were used to verify the urea dissociation concentration. The COIs of 55 false-positive sera were between 1.0 and 15.0; and 15 early HIV infection sera were between 1.0 and 20.0. All cases were confirmed by initial western blotting and follow-up western blotting. All cases were investigated epidemiologically.

**Figure 6:**
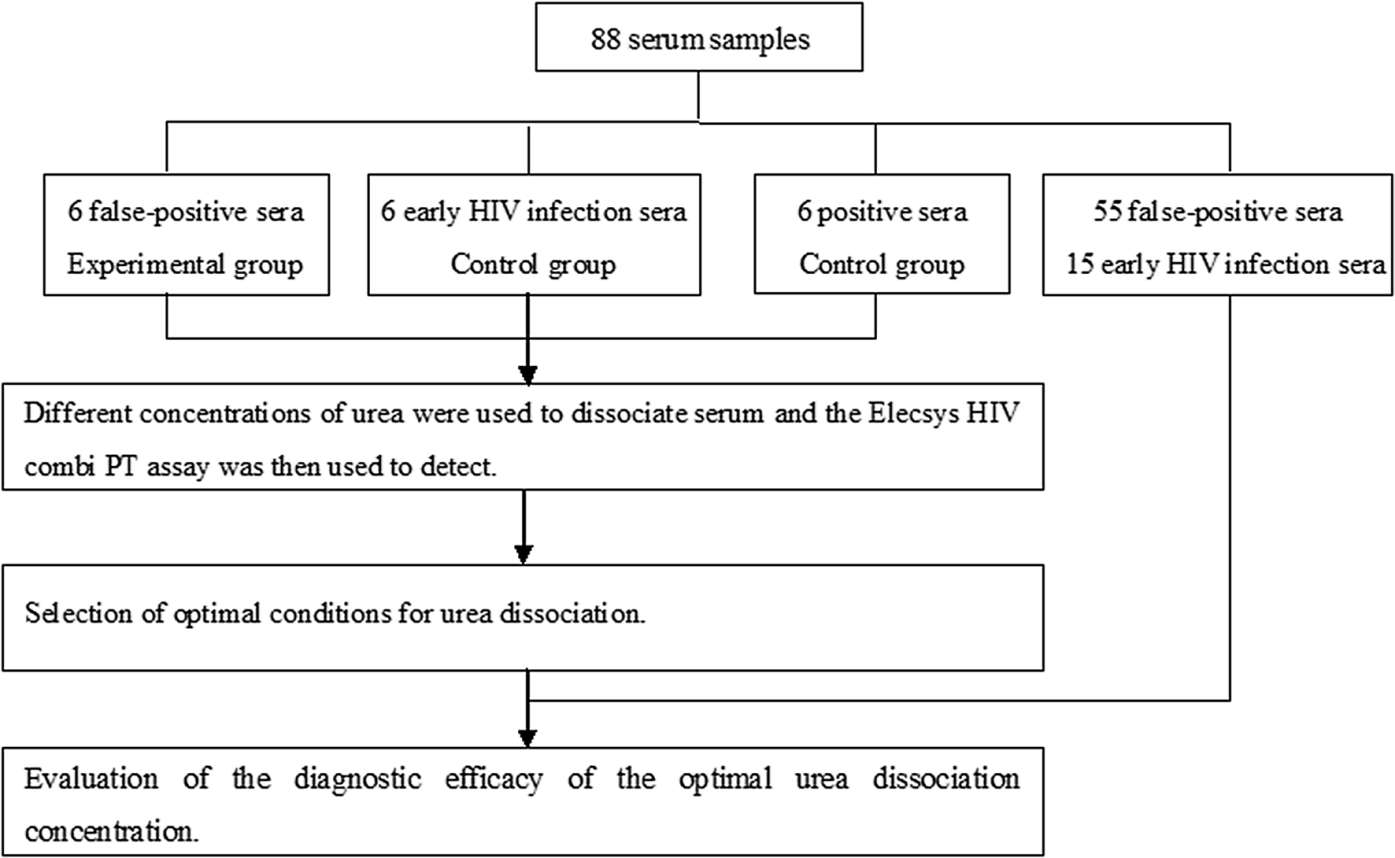
Flowchart depicting the study protocol.

88 serum samples were collected and cryopreserved at − 80 °C. All sera were thawed at room temperature and then centrifuged at 2583×*g* for subsequent tests. The research process is shown in the flow chart of Fig. 6 below.

The study protocol was approved by the Ethics Committee of the Affiliated Hospital of North Sichuan Medical College. All the experimental protocol and the methods were carried out in accordance with the relevant guidelines and regulations, and complied with the principles of the Declaration of Helsinki. As the samples included in the study were conducted anonymously, the ethics committee of the Affiliated Hospital of North Sichuan Medical College agreed to the exemption application of informed consent.

### Western blotting

Western blotting was used as the confirmatory test for HIV infection. Western blot HIV1/2 BLOT 2.2 (MP Biomedicals, Singapore) provides qualitative in vitro assays for class IgG antibodies against HIV 1 + 2 in serum or plasma for the diagnosis of HIV infection. The antibodies detected could be separated according to different HIV-1 gene product groups of env (gp120, gp160, gp41), gag (p17, p24, p39, p55), pol (p31, p51, p66), and HIV-2-specific antigen immobilized on the membrane. Western blotting was used as the gold standard in the study. All operations were carried out according to the manufacturer’s instructions. The results were interpreted according to the Chinese Centers for Disease Control and Prevention criteria, as follows ^6^: a positive result required the presence of at least two bands, including two env bands (HIV-1: gp41 and gp120/gp160 and HIV-2: gp36 and gp105/gp140) or one env band and one p24 band; an indeterminate result was defined as the presence of a band profile that did not meet the positive criteria; and a negative result was the absence of any specific bands.

### Urea dissociation test

Six cases of false-positive sera and twelve cases of positive control samples were used in urea dissociation tests to select the appropriate urea dissociation concentration. The specific steps are as follows: first, five sample tubes were taken and marked, then 0.4 mL serum was add into each tube; second, add the corresponding mass of urea into the sample tube, so that the serum urea concentration were 1 mol/L, 2 mol/L, 4 mol/L, 6 mol/L and 8 mol/L respectively; and lastly, Elecsys HIV combi PT assay were detected after allowing the urea to dissolve for 10 min. All operations were conducted according to the manufacturer’s instructions. The avidity index (AI) of sera was expressed by the ratio of the urea treated sample to the untreated sample; that is, AI = COI of urea dissociation/COI without urea dissociation.

### Verification experiment

Fifty-five cases of false-positive serum with a low COI and Fifteen early HIV infection serum samples were used in the verification experiment. The assay was carried out as follows. First, 0.5 mL serum was added into sample tube containing urea. After allowing the urea to dissolve for 10 min, Elecsys HIV combi PT assay were detected by electrochemiluminescence assays. The principle to set the critical value of AI was that the probability of early HIV infection being judged to be false negative was 0.01%. The specific steps are as follows: first, through the K-S test, the AIs of early HIV infection shows a normal distribution test; then, according to the function NORMDIST, the AI at 0.01% was calculated. The sensitivity and specificity of the best urea dissociation were then evaluated.

### Statistical analysis

The measurement data were expressed as the mean ± SEM, and the count data were expressed with percentage. The differences between the groups were assessed by one-way ANOVA with least significant difference (LSD) post-hoc analyses. All of the statistical analyses were performed using the Statistical Package for Social Sciences for Windows software, version 18.0 (SPSS Co., Inc., Chicago, IL). The statistical significance of all tests was defined as a P value of < 0.05 determined by two-tailed tests.

## Data Availability

All relevant data are within the manuscript and its Supporting Information files.
